# Alerting and Circadian Effects of Short-Wavelength vs. Long-Wavelength Narrow-Bandwidth Light during a Simulated Night Shift

**DOI:** 10.3390/clockssleep2040037

**Published:** 2020-11-25

**Authors:** Erlend Sunde, Torhild Pedersen, Jelena Mrdalj, Eirunn Thun, Janne Grønli, Anette Harris, Bjørn Bjorvatn, Siri Waage, Debra J. Skene, Ståle Pallesen

**Affiliations:** 1 Department of Psychosocial Science, Faculty of Psychology, University of Bergen, 5020 Bergen, Norway; anette.harris@uib.no (A.H.); staale.pallesen@uib.no (S.P.); 2 Department of Biological and Medical Psychology, Faculty of Psychology, University of Bergen, 5020 Bergen, Norway; torhild.pedersen@uib.no (T.P.); jelena.mrdalj@uib.no (J.M.); janne.gronli@uib.no (J.G.); 3 Department of Clinical Psychology, Faculty of Psychology, University of Bergen, 5020 Bergen, Norway; eirunn.thun@uib.no; 4 Department of Global Public Health and Primary Care, Faculty of Medicine, University of Bergen, 5020 Bergen, Norway; bjorn.bjorvatn@uib.no (B.B.); siri.waage@uib.no (S.W.); 5 Norwegian Competence Center for Sleep Disorders, Haukeland University Hospital, 5021 Bergen, Norway; 6 Chronobiology, Faculty of Health and Medical Sciences, University of Surrey, Guildford GU2 7XH, UK; d.skene@surrey.ac.uk; 7 Optentia Research Focus Area, North-West University, Vanderbijlpark 1900, South Africa

**Keywords:** short-wavelength light, night work, sleepiness, alertness, performance, circadian rhythm

## Abstract

Light can be used to facilitate alertness, task performance and circadian adaptation during night work. Novel strategies for illumination of workplaces, using ceiling mounted LED-luminaires, allow the use of a range of different light conditions, altering intensity and spectral composition. This study (ClinicalTrials.gov Identifier NCT03203538) investigated the effects of short-wavelength narrow-bandwidth light (λ_max_ = 455 nm) compared to long-wavelength narrow-bandwidth light (λ_max_ = 625 nm), with similar photon density (~2.8 × 10^14^ photons/cm^2^/s) across light conditions, during a simulated night shift (23:00–06:45 h) when conducting cognitive performance tasks. Light conditions were administered by ceiling mounted LED-luminaires. Using a within-subjects repeated measurements study design, a total of 34 healthy young adults (27 females and 7 males; mean age = 21.6 years, SD = 2.0 years) participated. The results revealed significantly reduced sleepiness and improved task performance during the night shift with short-wavelength light compared to long-wavelength light. There was also a larger shift of the melatonin rhythm (phase delay) after working a night shift in short-wavelength light compared to long-wavelength light. Participants’ visual comfort was rated as better in the short-wavelength light than the long-wavelength light. Ceiling mounted LED-luminaires may be feasible to use in real workplaces, as these have the potential to provide light conditions that are favorable for alertness and performance among night workers.

## 1. Introduction

At the beginning of this century it was established that humans have nonvisual photic input from a subset of intrinsically photosensitive retinal ganglion cells (ipRGCs) expressing the photopigment melanopsin, which is maximally sensitive to short-wavelength light [1,2,3]. These ipRGCs project directly to the main circadian pacemaker located in the hypothalamic suprachiasmatic nuclei (SCN), and this pacemaker controls and coordinates circadian rhythms [1,2]. Further insights have revealed that ipRGCs also project to various brain areas involved in sleep–wakefulness regulation, mood and even higher order cognitive processes [4,5,6]. Although melanopsin is the primary photopigment eliciting nonvisual effects, classical rods and cones also contribute to nonvisual responses to light via input to the ipRGCs [7,8].

Nonvisual responses sensitive to short-wavelength light include suppression of melatonin production [9,10], circadian phase shifting [11,12], pupil responses [13], and enhancement of alertness and performance [14,15,16]. It has been suggested that the alerting effects of light are strongest at night [5], when the circadian- and homeostatic drives for sleep are high, as postulated by the two-process model of sleep regulation [17]. Accordingly, several previous studies reporting alerting effects of short-wavelength narrow-bandwidth light [14,15,18] were conducted late in the evening or during the biological night, under relatively high circadian- and homeostatic sleep pressure. During the night, the alerting effects of short-wavelength light are induced by counteracting both the circadian and homeostatic drives for sleep, while during the day only the homeostatic sleep pressure is affected [19].

Especially at lower light intensity levels, short-wavelength narrow-bandwidth light (λ_max_ = 479 nm) was found to be more effective for eliciting subjective alerting responses than long-wavelength narrow-bandwidth light (λ_max_ = 627 nm), while at higher intensity levels the difference becomes less clear [20]. Note that long-wavelength narrow-bandwidth light (λ_max_ = 630 nm) also seems to be able to enhance alertness and performance compared to dark conditions [21,22]. Such findings have also been reported during daytime, as long-wavelength light improved alertness, assessed with electroencephalography, relative to darkness [23]. It was further noted that melatonin suppression is thus not needed for eliciting alerting effects in humans. Common for the previous narrow-bandwidth light studies were strict control of light exposure, and administration by special lighting set-ups such as custom-made light spheres [9,10,11,14,19,20], light goggles [15,21], light visors [16], or light boxes [22]. While the use of such specialized lighting set-ups allows for well controlled laboratory trials, the suitability in real-life settings may be limited.

Night work has consistently been associated with alertness and performance deterioration [24,25], and light interventions have the potential to counter these immediate effects of night work, both by its acute alerting properties [26] and via circadian phase shifting [27]. However, reviews of interventions to reduce the negative impact of night work (also chronic health effects) have indicated that definite conclusions on the beneficial effects of light interventions during night work cannot yet be drawn [28,29]. Recently, it was also noted that although most field studies indicate some beneficial effect of light during night work, methodological issues and diversity preclude conclusion about appropriate light schedules for night shift workers [30].

Due to nonvisual responses being sensitive to short-wavelength light, there has been an interest in employing short-wavelength enriched (i.e., blue-enriched) white light as a countermeasure against some of the negative impacts of night shift work. As such, recent studies have suggested beneficial effects of blue-enriched light on nocturnal alertness and performance [31,32,33]. On the other hand, an issue with the use of light interventions during night work, especially short-wavelength light, is the potential negative effects associated with light at night, e.g., melatonin suppression has been suggested as a mechanism for the increased risk of cancer among night shift workers [34,35]. Thus, studies have also investigated short-wavelength depleted/attenuated white light during simulated night shifts [36,37,38]. These studies have indicated that such lighting can reduce melatonin suppression and phase shifting of the melatonin rhythm, without having a negative impact on alertness and performance.

The development of light emitting diodes (LED) has made light characteristics such as spectral composition and light intensity easily controllable [39]. Thus, light exposures previously administered by specialized lighting set-ups, can now be administered via standard ceiling mounted LED-luminaires applicable for illumination of workplaces. However, only a few recent studies have used such LED-based lighting during simulated night work [33,40,41]. The effects of narrow-bandwidth lighting using ceiling mounted LED-luminaires during night work have not yet been investigated.

Most previous studies reporting alerting effects of short-wavelength narrow-bandwidth light have employed relatively low photon density [14,15,18,19], hence there is a lack of studies investigating narrow-bandwidth light levels that could be sufficient for a workplace setting. Three previous studies [14,15,19] used photon matching and a photon density of 2.8 × 10^13^ photons/cm^2^/s, comparing short-wavelength narrow-bandwidth light (λ_max_ = 460 nm) and medium-wavelength narrow-bandwidth light (λ_max_ = 550–555 nm) light. One study [18] compared short-wavelength narrow-bandwidth light (λ_max_ = 460 nm) and long-wavelength narrow-bandwidth light (λ_max_ = 640 nm), using an even lower photon density of 5 × 10^12^ photons/cm^2^/s (~1.0 lx). The latter study, however, was targeted towards work situations such as driving, where low intensity light would be practical.

The aim of the current study was to investigate how short-wavelength narrow-bandwidth light, compared to long-wavelength narrow-bandwidth light, administered by standard ceiling mounted LED-luminaires, affected alertness, task performance, and circadian adaptation during a simulated night shift. Similar photon density (~2.8 × 10^14^ photons/cm^2^/s) was used for both the short-wavelength (λ_max_ = 455 nm) and the long-wavelength (λ_max_ = 625 nm) narrow-bandwidth light, with photopic illuminances of 60.8 lx and 195.9 lx, respectively. Multiple measures were used to assess subjective alertness, mood states and task performance during the simulated night shift, as well as the magnitude of the circadian phase shift following the night shift. Limited constraints and requirements were put on the participants in order to mimic naturalistic working conditions as much as possible. We employed repeated measurements to assess the alertness dynamics during the night shift. We hypothesized that high photon density short-wavelength narrow-bandwidth light, administered by standard ceiling mounted LED-luminaires, would lead to better alertness, mood and performance during the night shift, compared to a night shift with long-wavelength narrow-bandwidth light. We also hypothesized that the night shift with short-wavelength light would lead to a larger phase delay of the circadian rhythm, compared to a night shift with long-wavelength light. In addition, we investigated participants’ subjective evaluation of the lighting conditions, as well as visual comfort, during the night shift.

## 2. Materials and Methods

### 2.1. Study Design

**Figure 1:**
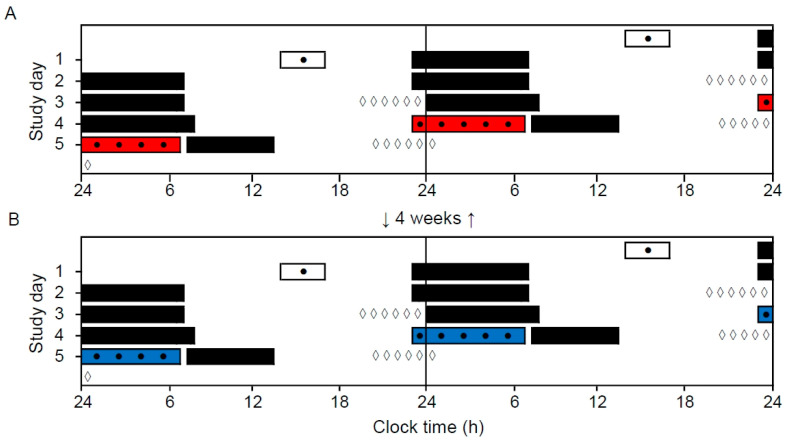
Double-raster plot of the simulated night shift protocol. The protocol included two simulated night shifts (from 23:00 to 06:45 h) performed in a laboratory with long-wavelength narrow-bandwidth light (**A**) and short-wavelength narrow-bandwidth light (**B**). The night shifts were separated by 4 weeks and the order of conditions was counterbalanced. White bars indicate enrollment and practice session (before the first night shift only) in the laboratory. Black bars indicate assumed sleep periods at home. Colored bars indicate night shifts in the laboratory. Black dots indicate primary test bouts including the Positive and Negative Affect Schedule (PANAS), the Karolinska Sleepiness Scale (KSS), a Psychomotor Vigilance Task (PVT), and a Digit Symbol Substitution Test (DSST). White diamonds indicate salivary dim-light melatonin sampling at home.

A within-subject repeated measures design was applied to investigate the effects of narrow-bandwidth light exposure (short-wavelength vs. long-wavelength) during two simulated (laboratory) night shifts (Figure 1). Participants came to the laboratory, in groups of four to eight participants, on two separate evenings to complete the night shift (23:00–06:45 h) (short-wavelength and long-wavelength light counterbalanced). The simulated night shift sessions were separated by 4 weeks. The study was conducted at a high latitude (~60° N), and during a time of year (October 2018 to March 2019) with limited daylight exposure, in order to accentuate effects of light exposure.

### 2.2. Participants

Thirty-four young adults participated in the study (27 females and 7 males; mean age 21.6 years, SD = 2.0 years, range 18–27 years). Six participants dropped out after the first shift, and two had their second night shift cancelled as their group (*n* = 2) was considered too small to carry out the night shift. Thirty-one (6 males) and 29 participants (7 males) completed the night shift in short-wavelength and long-wavelength light, respectively. Overall, 26 participants (6 males: mean age 21.6 years, SD = 1.9 years) completed both night shifts. A power analysis was conducted a priori. Expecting a medium effect size (Cohen’s *d* = 0.5) with significance level of 0.05, power of 0.8 and correlation among repeated measures (*n* = 5) of 0.5 in a repeated measures within factors design (ANOVA), 21 participants were calculated to be needed [42]. The sample size complied also with the recently recommended sample size (paired *t*-test; *n* = 26) for studies investigating the alerting effects of light [43].

Participants were recruited among university students by invitation via the learning platform and/or mass e-mail. Prior to enrollment, subjects completed an online screening survey to ensure eligibility. All participants had good to excellent self-reported health and body mass index <30 kg/m^2^. Participants reported no current or relevant history of psychiatric-, neurological-, cardiovascular-, lung-, sleep- and/or eye diseases/disorders, and normal color vision (also measured with the 17-plate Ishihara Test for Color Deficiency). Participants were not on medications (except some females were on oral contraceptives) and females were not pregnant or breastfeeding. None of the participants were extreme chronotypes according to the short Morningness–Eveningness Questionnaire [44], and none were engaged in night work and/or had transmeridian travel in the month prior to or during the study period. All recruited participants reported habitual sleep duration of 6–10 h per night, with habitual wake times between 06:00 and 10:00 h. These sleep criteria were set to reduce variation in participants’ circadian phase, and homeostatic sleep pressure. Furthermore, the sleep criteria ensured that the participants performed the night shifts during their biological night. Adherence was confirmed by sleep diaries and wrist-actigraphy (Actiwatch 2 or Actiwatch Spectrum; Philips Respironics, The Netherlands) for three days prior to each night shift. The light sensor on the Actiwatch was also used to assess light exposure in the hours preceding the night shifts (see Table S1). Participants refrained from alcohol use three days prior to and during each night shift, caffeine use in the period from 10:00 h on the day prior to (i.e., morning coffee was allowed) and during each night shift, and tobacco/nicotine use at least 2 h prior to and during each night shift.

### 2.3. Procedures

Three days prior to the first night shift, eligible subjects were invited to an enrollment session. All subjects signed an informed consent form and completed a set of questionnaires and practiced the performance tasks used in the experiment. Standard illumination (~4000 K) of approximately 200 lx at eye level (vertical plane, 120 cm height) was applied during the enrollment session. In addition, participants received the actigraph and equipment for collecting saliva samples (instructions sheet, dark sunglasses, and saliva tubes) at home. The participants slept at home with no restrictions on activities or light exposure, except during saliva sampling in the evening on the day before the night shift, and no napping was allowed on the day prior to the night shift.

The simulated night shift and light exposure (short-wavelength or long-wavelength light) started at 23:00 h and ended at 06:45 h. The first 30 min were used for adaptation and preparation, including completing questionnaires assessing visual comfort (headache and eye strain symptoms), and evaluation of the lighting conditions. At 23:30 h, the first of five main test bouts commenced, and was repeated every 90 min at 01:00, 02:30, 04:00 and 05:30 h, respectively. One test bout lasted ~20 min and included the Positive and Negative Affect Schedule (PANAS), the Karolinska Sleepiness Scale (KSS), a 10-min computerized Psychomotor Vigilance Task (PVT), and a 2-min computerized Digit Symbol Substitution Test (DSST). During testing, participants were seated at their desk space and wore noise cancelling headsets (BOSE QuietComfort 25, BOSE Corp., Framingham, MA, USA). Between the main test bouts, other tests and questionnaires were administered, such as a pegboard test, working memory, reversal learning, numerosity discrimination, and a Go/No-go test, as well as a questionnaire on evaluation of moral issues. In this paper, we report results from the main test bouts only, in addition to visual comfort and evaluation of the lighting conditions. The protocol was the same during both night shifts. Participants had several short breaks (usually 10–15 min) allowing quiet activities (e.g., reading and talking). Participants remained seated at their designated desk space for most of the time during the whole shift, except for toilet breaks, for which they had to walk through a dimly lit hallway. A researcher was present in the laboratory during the whole shift to ensure completion of tasks and adherence to the protocol. A standardized meal/snack (~200 kcal) was provided at about 02:00 and 05:00 h, and water was available ad libitum. No other foods or drinks were allowed.

Participants completing the study were compensated for their participation and inconvenience. The study was conducted in accordance with the Declaration of Helsinki, and the protocol was approved by the Norwegian Regional Committee for Medical and Health Research Ethics, health region West (No. 2016/1903). The study was preregistered, with ClinicalTrials.gov Identifier NCT03203538.

### 2.4. Laboratory and Light Exposure

**Figure 2:**
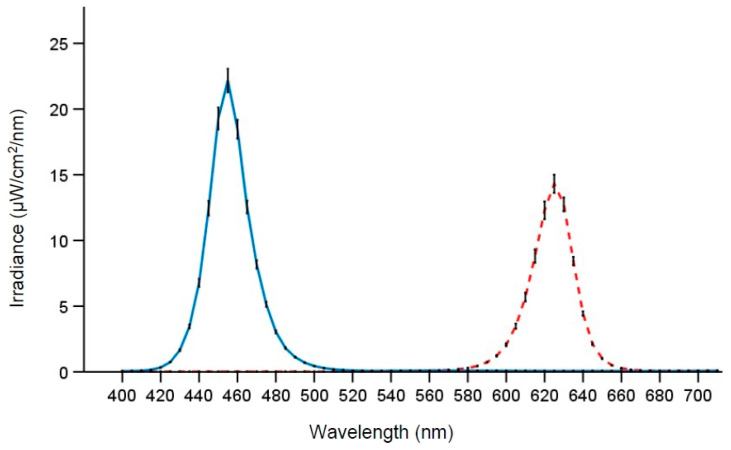
Spectral distribution of the short-wavelength narrow-bandwidth light (solid line) and the long-wavelength narrow-bandwidth light (dotted line). Means and SD (error bars) for measurements (vertical plane) at eye level.

The laboratory (30 m^2^) had no windows, was air-conditioned and temperature maintained at ~22 °C. Participants were designated to one of eight similar desk spaces, separated by partition walls, with equivalent desktop computers. Computer screens were fitted with a filter foil (Metolight SFG-10; Asmetec, Kirchheimbolanden, Germany) that blocked all light wavelengths <520 nm. The room was equipped with 20 ceiling mounted LED-luminaires (Modul R 600 LED CCT/RGB MP; Glamox Luxo Lighting AB, Gothenburg, Sweden; size 60 × 60 cm), providing uniform illumination of the room. The light conditions were measured at the beginning, middle, and end of each night shift, at two desk spaces, one on each side of the room. Measurements were performed at eye level (vertical plane, 120 cm height) while seated at the desk space, using a spectroradiometer (GL Spectics 1.0 T; GL Optic, Puszczykowo, Poland). Lighting parameters (Table 1) were calculated according to the CIE S 026 Toolbox—version 1.049 [45]. The photon density was similar for the two light conditions, and both light conditions had <15 nm half-peak bandwidth (Figure 2). Note that participants’ posture and gaze direction were not strictly controlled (except when engaged in the performance tasks). The light levels thus represent the approximate light exposure at eye level during most of the time in the laboratory.

The stability of the light exposure (irradiance) during the night shifts was analyzed using a linear mixed model with Group (six groups of participants) included as a random factor and Light (long-wavelength vs. short-wavelength), Time (beginning, middle or end of shift), and the interaction Light by Time entered as fixed factors. There was a significant effect of Light (F_1,54_ = 1087.44; *p* < 0.001) with higher irradiance of the short-wavelength light (EMM = 125; SE = 1 µW/cm^2^) compared to long-wavelength light (EMM = 82; SE = 1 µW/cm^2^), but there were no significant effects of Time (F_2,54_ = 1.07; *p* = 0.351) or the Light by Time (F_2,54_ = 0.27; *p* = 0.765) interaction.

### 2.5. Measures

#### 2.5.1. Mood and Subjective Alertness

Positive and negative mood were measured with PANAS [46]. PANAS comprises 20 items/words describing different feelings and emotions, and participants indicated to which extent they felt a certain way at that moment on a 5-point Likert scale ranging from 1, “very slightly or not at all”, to 5, “extremely”. PANAS was completed at the beginning of each main test bout, and the positive and negative affect subscales showed internal reliability of Cronbach’s *α* = 0.92 and Cronbach’s *α* = 0.68, respectively.

Subjective alertness/sleepiness was measured with KSS [47]. KSS comprises a 9-point Likert scale ranging from 1, “very alert”, to 9, “very sleepy, fighting sleep, strenuous to keep awake”. The KSS was completed at the beginning and at the end of each main test bout, and the average KSS rating for each test bout was analyzed.

#### 2.5.2. Task Performance

Participants’ vigilance and ability to sustain attention was assessed with a 10 min PVT [48,49]. The PVT is sensitive for detecting effects of sleep deprivation and shows minor aptitude and learning effects [48,49]. Participants monitored a rectangle/box on the computer screen and responded with their dominant hand by pressing the space bar when a visual stimulus (a counting timer) appeared inside the box. After each trial, a 1 s feedback on response time (RT) was provided. The interstimulus interval randomly varied from 2 to 10 s including feedback. If no response was registered after 30 s (treated as a valid trial with RT = 30,000 ms), a sound alerted the participant and a new trial began. RTs shorter than 100 ms were considered as false starts. For all PVTs performed during the night shifts (*n* = 300), the mean number of trials per test was 95 (SD = 6). The outcome measures reported here are the “mean 1/RT” (reciprocal RTs), “lapses” (number of RTs ≥ 500 ms) and the “mean RT500” (mean RTs with lapses excluded).

Participants also performed a 2-min DSST [50], which was administered directly following the PVT. The DSST is sensitive to changes in cognitive function, and able to detect sleep deprivation effects [50]. DSST performance improves, however, with repeated administration [50], and to minimize such learning effects the DSST was practiced once during the enrollment session, and the symbol–digit pairs were randomized across administrations. A target symbol (one of nine symbols presented in a random order) was presented at the center of the screen and had to be paired with the corresponding digit from a symbol–digit array shown at the bottom of the screen. The mouse pointer was used for selecting digits and if no response was recorded after 5 s, the next trial began. The mean number of trials per test was 76 (SD = 8), and the outcome measure derived was the “*n* correct” (number of correct responses).

#### 2.5.3. Visual Comfort and Evaluation of Lighting Conditions 

Visual comfort was assessed with the Headache and Eye Strain Scale (HES) [51] at the beginning (23:15 h), middle (03:15 h), and at the end (06:15 h) of each night shift. The HES questionnaire comprises 8 items/symptoms: “irritability”, “headache”, “eye strain”, “eye discomfort”, “eye fatigue”, “difficulty focusing”, “difficulty concentrating”, and “blurred vision”. Participants indicated to what extent they currently experienced the symptoms on a 4-point scale (1 = absent, 2 = slight, 3 = moderate, and 4 = severe).

Participants’ subjective evaluation of the lighting conditions was assessed using a 7-point semantic differential scale questionnaire adapted from [52,53]. The questionnaire was completed at the beginning (23:15 h) and at the end (06:30 h) of each night shift. Four items comprised the subscale “pleasantness” of the lighting (“unpleasant–pleasant”, “uncomfortable–comfortable”, “disturbing–not disturbing” and “causing glare–not causing glare”) which showed good reliability with Cronbach’s *α* = 0.87. Four single items assessed the “clearness” (“unclear–clear”), “color” (“warm–cold”), “brightness” (“dim–bright”), and whether the lighting was “activating” (“relaxing–stimulating”). One item probed whether the light was “suitable for work” (“unsuitable for work–suitable for work”). For each of the six outcomes, the average rating for the two time points was used for analysis, as there were no differences between time of assessment.

#### 2.5.4. Circadian Phase

Participants’ circadian phase was assessed before and after each night shift by measuring salivary dim light melatonin onset (DLMO). Baseline DLMO was sampled in the evening on the day before each night shift, while the final DLMO was sampled in the evening on the day after each night shift. Participants provided hourly saliva samples at home (six samples each evening), using Salivette tubes (Sarstedt AG & CO, Nümbrecht, Germany). Sampling of baseline DLMO started 4 h before and lasted until 1 h after the participants’ usual bedtime. Relative to baseline DLMO sampling, the time of final DLMO sampling was delayed by 1 h (hence the last sampling was 2 h after usual bedtime). A similar protocol as described in a previous study was applied [54]. To ensure dim light during sampling, participants wore dark sunglasses (Uvex Athletic ISO 9001, Uvex Winter Holding GmbH & Co., Fürth, Germany; and/or Uvex Genesis S3208 Infra-dura 5.0, Honeywell, Charlotte, NC, USA) from 1 h before and during the whole sampling period. These glasses’ lenses reduce light intensity to <1% [54]. Before delivery to the laboratory for storage at −70 °C, participants were instructed to label the samples with clock time and store them in their domestic refrigerator (4 °C).

The saliva samples were assayed with an enzyme-linked immunosorbent assay kit (EK-DSM, Bühlman Laboratories, Schönenbuch, Switzerland) with a detection limit of 0.5 pg/mL and a functional sensitivity of 1.6–20.5 pg/mL. A Wallac 1420 Multilabel counter (Perkin Elmer Inc., Waltham, MA, USA) was used to analyze the samples. The inter-assay variation was 18.4% and 14.8% for the low and high quality control, respectively. The mean (SD) melatonin value was 4.4 (0.8) and 13.7 (2.0) pg/mL for the low and high quality control, respectively. The DLMO was set as the clock time when salivary melatonin concentration reached 4 pg/mL, using linear interpolation between adjacent samples [55]. If melatonin concentration during sampling reached 3 pg/mL, but not 4 pg/mL, linear extrapolation was used. The magnitude of the phase shift was calculated as the difference between baseline DLMO and final DLMO for each individual. As an estimate of the temperature minimum (Tmin), 7 h was added to the baseline and final DLMO [27].

Due to missing DLMO data, the circadian phase shift could not be calculated for all participants. After the night shift in long-wavelength light, phase shifts were estimated for 22 (76%) of 29 participants, while after the night shift in short-wavelength light, phase shifts were estimated for 19 (61%) of 31 participants. For the long-wavelength light condition, one participant did not reach 3 pg/mL during final sampling, four had melatonin levels exceeding 4 pg/mL for all samples at baseline and/or final DLMO sampling, and two participants did not provide saliva samples. For the short-wavelength light condition, three participants did not reach 3 pg/mL during final sampling, eight had melatonin levels exceeding 4 pg/mL for all samples at baseline and/or final DLMO sampling, and one did not perform the final sampling.

### 2.6. Statistical Analysis

Participant characteristics were described by means and standard deviations (SD). To assess the effects of light exposure, Linear Mixed Model (LMM) analyses and Generalized Linear Mixed Model (GLMM) analyses were performed. For positive mood, negative mood, KSS, PVT mean 1/RT and mean RT500, and the DSST *n* correct (separate analyses per variable), three LMM models, using maximum likelihood estimation, were performed for each of the variables: (1) A random effect model with Participant included as a random effect, (2) a main effects model with Light (short-wavelength vs. long-wavelength) and Time (23:30 vs. 01:00, 02:30, 04:00 and 05:30 h) entered in the model as fixed factors, and (3) an interaction effect model with the Light by Time interaction also included as a fixed factor. For all variables, the main effects model had a better model fit, as assessed with a Likelihood Ratio Test (LRT), than the random effect model. For variables with a significant Light by Time interaction, the LRT indicated that the interaction effect model had the best model fit. The LRT was performed by comparing the difference in −2 times the log of the likelihood between successive models following a chi-square distribution, using degrees of freedom equal to the difference in the number of parameters between the compared models. The normality of the residuals from the interaction effect models were assessed with Shapiro–Wilk tests and normality plots to confirm that assumptions were met. Post-hoc comparisons were conducted using Bonferroni corrections, and the estimated marginal means (EMM) and standard errors (SE) are reported. R-squared (*R*^2^) was calculated representing the proportion of reduction in variance of the residuals (this measure can also have negative values), and *R*^2^ for the interaction effect models are reported.

PVT lapses were analyzed using GLMM models, with a negative binominal distribution, as this is a count variable showing overdispersion. A similar procedure as described above (random-, main- and interaction effect models) was applied. The LRT approach for assessing model fit is not appropriate for the GLMM analyses, as restricted maximum likelihood estimation is used. Instead, the Akaike’s information criterion (AIC) and the Schwarz’s Bayesian criterion (BIC) were used for comparison of model fit (smallest values were preferred), and accordingly, the interaction effects model had the best fit.

The HES symptoms, and the items probing evaluation of the lighting conditions, were analyzed using LMMs as described above. However, there were only three time points (23:15, 03:15 and 06:15 h) for the HES’ Time factor, and for evaluation of lighting conditions there was no time factor (nor interaction) entered into the LMM models.

The magnitude of the phase shift was analyzed using LMMs with Participant as a random effect, and Light (short-wavelength vs. long-wavelength) included as a fixed factor, employing similar settings and procedures as described above. To investigate whether there were differences in baseline DLMO between the conditions, paired samples *t*-tests were used. Pearson’s product–moment correlation coefficients were calculated to assess whether baseline DLMO was related to the phase shift magnitude.

Statistical analyses were performed using IBM SPSS Statistics, version 25 (IBM Corp., Endicott, NY, USA). 

## 3. Results

### 3.1. Mood and Subjective Alertness

#### 3.1.1. PANAS: Effects on Mood

**Figure 3:**
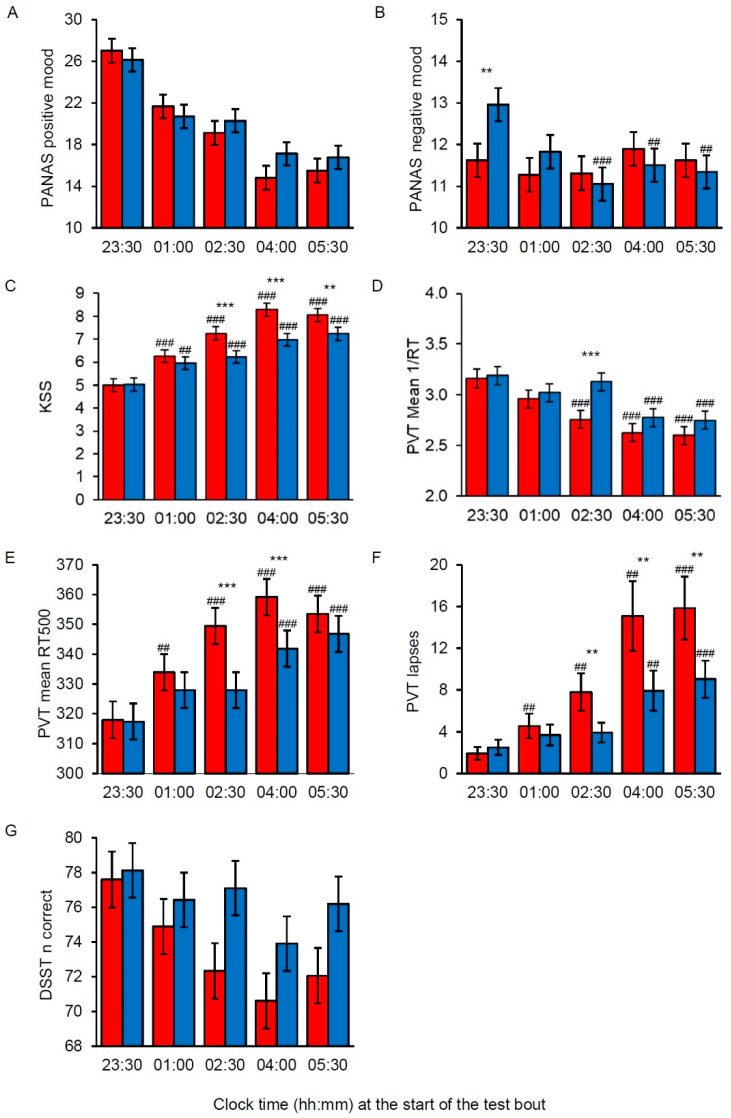
Mood, sleepiness and performance during a simulated night shift in long-wavelength narrow-bandwidth light (red bars) and short-wavelength narrow-bandwidth light (blue bars). The bars represent estimated marginal means with error bars indicating standard error. (**A**) Positive mood assessed with the Positive and Negative Affect Schedule (PANAS). (**B**) Negative mood assessed with PANAS. (**C**) Subjective sleepiness assessed with the Karolinska Sleepiness Scale (KSS). (**D**) Reciprocal response times (mean 1/RT) on the Psychomotor Vigilance Task (PVT). (**E**) RTs excluding lapses (mean RT500) on the PVT. (**F**) Number of lapses (RTs ≥ 500 ms) on the PVT. (**G**) Number of correct responses on the Digit Symbol Substitution Test (DSST). Significant differences indicated for variables with a significant Light by Time interaction only. Number symbols (#) indicate significant difference compared to the first test bout (23:30 h), and asterix symbols (*) indicate significant difference between light conditions. ##; ** = *p* < 0.01, ###; *** = *p* < 0.001.

Analyses of positive mood and negative mood indicated no significant main effects of Light, but there was a significant main effect of Time for both measures (Table 2). Positive mood was reduced at 01:00, 02:30, 04:00 and 05:30 h (*p* < 0.001, all) compared to 23:30 h. Negative mood was reduced at 02:30 h (*p* = 0.002) compared to 23:30 h. For positive mood, there was no significant Light by Time interaction effect (Figure 3A). For negative mood, there was a significant Light by Time interaction effect, and post-hoc comparisons revealed increased negative mood in short-wavelength light at 23:30 h (Figure 3B).

#### 3.1.2. KSS: Effects on Subjective Sleepiness

For KSS, there were significant main effects of Light with reduced sleepiness in short-wavelength light, and Time with increased sleepiness at 01:00, 02:30, 04:00 and 05:30 h (*p* < 0.001, all) compared to 23:30 h (Table 2). There was also a significant Light by Time interaction effect, with post-hoc comparisons showing reduced sleepiness in short-wavelength light in the middle and later parts of the night shift (Figure 3C).

### 3.2. Task Performance

#### 3.2.1. PVT: Effects on Sustained Attention

Analysis of both mean 1/RT and mean RT500 revealed significant main effects of Light with faster RTs in short-wavelength light, and Time with slower RTs at 01:00 (mean 1/RT: *p* < 0.001; mean RT500: *p* = 0.005), 02:30, 04:00 and 05:30 h (*p* < 0.001, all) compared to 23:30 h (Table 2). There were also significant Light by Time interaction effects, and post-hoc comparisons revealed faster RTs in short-wavelength light in the middle and later parts of the night shift (Figure 3D,E). Results were similar for the number of lapses, with significant main effects of Light with fewer lapses in short-wavelength light, and Time with more lapses at 01:00 (*p* = 0.002), 02:30 (*p* = 0.001), 04:00 and 05:30 h (*p* < 0.001, both) compared to 23:30 h (Table 2). There was also a significant Light by Time interaction effect, with post-hoc comparisons showing fewer lapses in short-wavelength light in the middle and later parts of the night shift (Figure 3F).

#### 3.2.2. DSST: Effects on Number of Correct Responses

For the DSST n correct, there were main effects of Light with more correct responses in short-wavelength light, and Time with fewer correct responses at 02:30 (*p* = 0.018), 04:00 (*p* < 0.001) and 05:30 h (*p* = 0.002) compared to 23:30 h (Table 2). There was no significant Light by Time interaction effect for the DSST (Figure 3G).

### 3.3. Visual Comfort and Evaluation of Lighting Conditions

#### 3.3.1. Visual Comfort: Effects on Headache and Eye Strain Symptoms

**Figure 4:**
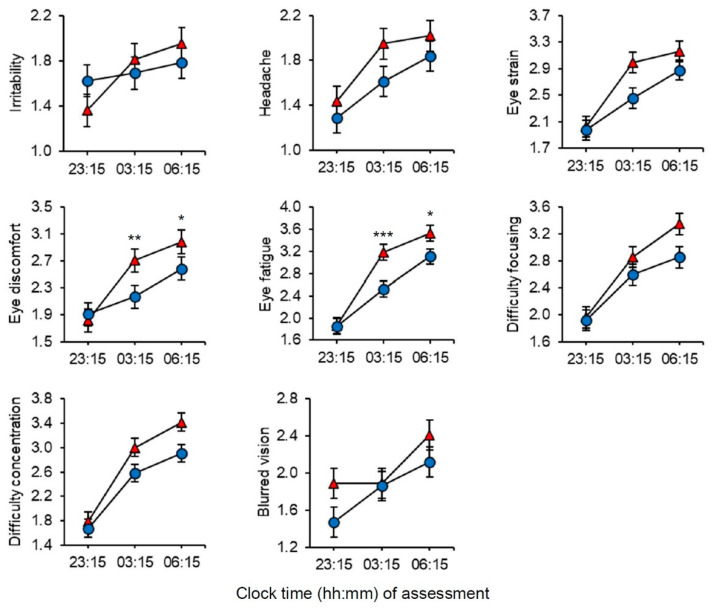
Visual comfort assessed with the headache and eye strain scale (1–4 (severe)) during a simulated night shift in long-wavelength narrow-bandwidth light (triangles) and short-wavelength narrow-bandwidth light (circles). Data points are the estimated marginal means with error bars indicating standard error. Significant differences between light conditions indicated for variables with a significant Light by Time interaction only. * = *p* < 0.05, ** = *p* < 0.01, *** = *p* < 0.001.

There were significant main effects of Light for all symptom categories, except irritability, with reduced symptoms in short-wavelength light (Table 2). For all categories there were significant main effects of Time with increased symptoms at 03:15 h (irritability: *p* = 0.032; blurred vision: *p* = 0.263; all other *p* < 0.001) and at 06:15 (*p* ≤ 0.001, all) compared to 23:15 h. For eye discomfort and eye fatigue, there were significant Light by Time interaction effects, and post-hoc comparisons showed reduced symptoms in short-wavelength light in the middle and late parts of the night shift (Figure 4). There were no significant Light by Time interaction effects for irritability, headache, eye strain, difficulty focusing, difficulty concentrating or blurred vision (Figure 4).

#### 3.3.2. Subjective Evaluation of the Lighting Conditions

Evaluation of the lighting conditions revealed a significant main effect of Light for color (F_1,60_ = 124.89; *p* < 0.001; *R*^2^ = 0.68), with short-wavelength light (EMM = 5.58, SE = 0.20) evaluated as colder than long-wavelength light (EMM = 2.43, SE = 0.20), brightness (F_1,60_ = 8.78; *p* = 0.004; *R*^2^ = 0.13), with short-wavelength light (EMM = 4.60, SE = 0.23) evaluated as brighter than long-wavelength light (EMM = 3.64, SE = 0.23), and whether the light was activating (F_1,60_ = 13.56; *p* < 0.001; *R*^2^ = 0.18), with short-wavelength light (EMM = 4.55, SE = 0.17) evaluated as more activating than long-wavelength light (EMM = 3.64, SE = 0.18). Note that for these variables, models were run without the random intercept as an error showed that the Hessian matrix was not positive definite, suggesting redundant covariance parameters. There was no significant main effect of Light for pleasantness (F_1,30_ = 1.31; *p* = 0.262; *R*^2^ = 0.01), with similar estimates for short-wavelength light (EMM = 4.09, SE = 0.22) and long-wavelength light (EMM = 3.81, SE = 0.21), clearness (F_1,31_ = 0.00; *p* = 0.953; *R*^2^ < 0.01), with similar estimates for short-wavelength light (EMM = 4.15, SE = 0.20) and long-wavelength light (EMM = 4.14, SE = 0.20), and whether the lighting was considered suitable for work (F_1,29_ = 2.50; *p* = 0.125; *R*^2^ = −0.26), with similar estimates for short-wavelength light (EMM = 3.75, SE = 0.26) and long-wavelength light (EMM = 3.38, SE = 0.26).

### 3.4. Circadian Phase

**Figure 5:**
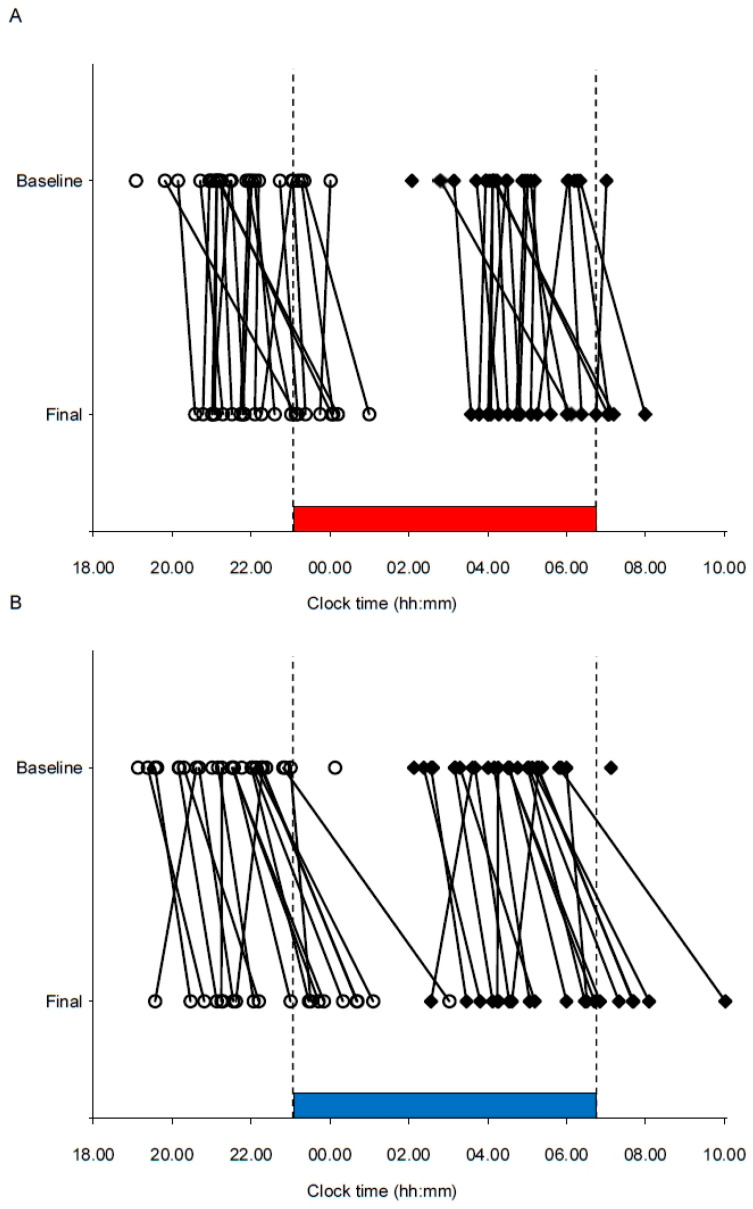
Phase markers for individual participants before (Baseline) and after (Final) a simulated night shift in (**A**) long-wavelength narrow-bandwidth light and (**B**) short-wavelength narrow-bandwidth light. Open circles indicate salivary dim light melatonin onset (DLMO) for each participant. Filled diamond squares indicate estimated temperature minimum (DLMO + 7 h) for each participant. Lines are drawn between the baseline and final markers for each participant. The vertical dotted lines and colored bars indicate the start and end times of the night shift and light exposure (23:00–06:45 h).

For long-wavelength light, baseline DLMO (*n* = 26; mean = 21:42 h, SD = 1:09 h) ranged from 19:05 to 00:01 h, and final DLMO (*n* = 22; mean = 22:25 h, SD = 1:15 h) ranged from 20:35 to 01:00 h (Figure 5A). For short-wavelength light, baseline DLMO (*n* = 27; mean = 21:23 h, SD = 1:13 h) ranged from 19:08 to 00:08 h, and final DLMO (*n* = 20; mean = 22:46 h, SD = 1:49 h) ranged from 19:34 to 03:02 h (Figure 5B). There was no significant difference in baseline DLMO for the 22 participants with complete baseline DLMO estimates (short-wavelength light: mean = 21:36 h, SD = 1:09 h, long-wavelength light: mean = 21:46 h, SD = 1:14 h; *t*_21_ = 0.91; *p* = 0.375). A similar result was found when analyzing baseline DLMO for the 12 participants with complete baseline and final DLMO estimates in both light conditions (short-wavelength light: mean = 21:26 h, SD = 0:59 h; long-wavelength light: mean = 21:41 h, SD = 1:11 h, *t*_11_ = 1.03; *p* = 0.324). 

For circadian phase shift, there was a significant main effect of Light (F_1,24_ = 5.33; *p* = 0.030; R^2^ = 0.11) indicating larger phase shift (delay) after a night shift in short-wavelength light (EMM = 1:26 h, SE = 0:16 h) compared to long-wavelength light (EMM = 0:36 h, SE = 0:15 h). There was no significant correlation between the magnitude of the phase shift and baseline DLMO for either short-wavelength (*r* = 0.33; *p* = 0.167) or long-wavelength (*r* = −0.33; *p* = 0.131) light.

## 4. Discussion

In this study, we investigated the effects of nocturnal short-wavelength narrow-bandwidth light (λ_max_ = 455 nm) compared to long-wavelength narrow-bandwidth light (λ_max_ = 625 nm) with similar photon density (~2.8 × 10^14^ photons/cm^2^/s), administered by standard ceiling mounted LED-luminaires, during a simulated night shift. To our knowledge, this study is the first to employ such LED-based light conditions during night work. As expected, subjective sleepiness increased, positive mood was reduced and task performance deteriorated during the night shift in both light conditions. However, with short-wavelength light the increase in sleepiness and the deterioration of task performance across the night shift was less severe than with long-wavelength light. Overall, our hypothesis of better alertness and performance during a night shift with short-wavelength narrow-bandwidth light, compared to long-wavelength narrow-bandwidth light, was supported. We did, however, not find beneficial effects of short-wavelength light on participants’ mood states. Participants’ melatonin onset was mainly phase delayed after the night shifts, and there was a significantly larger phase delay after the night shift with short-wavelength light compared to long-wavelength light. Hence, our hypothesis of a greater phase delay with short-wavelength narrow-bandwidth light was supported. There were no statistically significant differences between the light conditions in terms of participants’ evaluation of its pleasantness and suitability for work. However, we found evidence of improved visual comfort with short-wavelength light compared to long-wavelength light.

The results on subjective sleepiness and task performance showed very similar patterns, with beneficial effects of short-wavelength light emerging from 02:30 h onwards. PVT performance was better in short-wavelength light, both in terms of generally faster RTs (mean 1/RT), faster RTs in the optimal domain (RT500), and fewer attentional lapses (i.e., slow RTs) than in long-wavelength light. A similar pattern was observed for DSST performance, albeit no statistically significant interaction effect was found. Previous studies have reported beneficial effects of late evening and nocturnal exposure to short-wavelength light on alertness level and/or task performance [14,15,18]. However, as discussed in the introduction, those studies employed substantially lower photon densities than the current study. As noted, the previous studies were highly controlled laboratory trials using special lighting set-ups. The current results thus add to previous findings showing that short-wavelength narrow-bandwidth light, administered by standard ceiling mounted LED-luminaires in a naturalistic setting, also elicits alerting and performance enhancing responses, compared to long-wavelength narrow-bandwidth light. In addition, the results indicate that these effects can be achieved using higher photon densities than previously reported. As noted in the introduction, long-wavelength light may also elicit alerting responses compared with dark conditions. In the afternoon, long-wavelength narrow-bandwidth light (λ_max_ = 630 nm) improved alertness, while short-wavelength narrow-bandwidth light (λ_max_ = 470 nm) did not improve alertness, compared to darkness [23]. These findings do not match the alerting effect of nocturnal short-wavelength light, compared to long-wavelength light, in the present study, likely due to the role of melatonin suppression during nighttime. We did not have an additional night shift in dim light or in standard light conditions, hence the alerting effects of our light conditions cannot be compared to dim light or standard light conditions.

Regarding mood states, the positive mood indicator showed no statistically significant difference between the light conditions. For negative mood, there was a significantly higher score in short-wavelength light compared to long-wavelength light on the first assessment at 23:30 h, a difference that vanished in the subsequent sessions. It is not clear why this difference occurred, but it is possible that exposure to short-wavelength light initially had a negative impact on mood, and that longer than 30 min adaptation to the light is needed to reduce this effect. We did not assess mood prior to the light exposure. Hence, it is possible, yet unlikely, that there was a difference in negative mood prior to the night shift between the two light conditions. Thus, we cannot rule out that the short-wavelength light actually reduced the negative mood score compared to the long-wavelength light.

In terms of circadian phase, the significantly larger phase delay observed with short-wavelength light shows that overall, the participants’ circadian rhythm became more strongly entrained to night work after a night shift in short-wavelength light compared to long-wavelength light. However, the individual differences in circadian responses indicate that factors other than the light are also at play, such as differences in sensitivity to light [56]. Notably, following the night shift, the melatonin rhythm of a few participants showed the opposite phase shifting response (i.e., phase advance), although they had a similar initial melatonin onset time. Since the light exposure in the current study was kept constant during the whole shift, most participants were also exposed to light after their estimated Tmin, in the phase advance part of the participants’ phase response curves (PRC) to light [57,58]. As circadian responses are sensitive to short-wavelength light, short-wavelength light exposure after Tmin may have attenuated the phase delay to a larger degree than long-wavelength light, and/or caused a phase advance of the melatonin rhythm. Nevertheless, the current findings are in line with previously reported data showing a greater phase delay with short-wavelength narrow-bandwidth light administered before Tmin [11]. Individual differences in phase shifting responses to light have also been noted previously [11], and a recent study found that variations in the distribution of daily light exposure relative to the PRC accounted for a large portion of the variable rates of circadian adaptation among real night workers [59]. 

Participants evaluated the short-wavelength light condition as colder, brighter, and more activating than the long-wavelength light. The opinion of the short-wavelength light being brighter seems somewhat remarkable, as the photopic illuminance of the long-wavelength light (195.9 lx) was substantially higher than the short-wavelength light (60.8 lx). Nevertheless, similar findings were reported in a study comparing brightness experiences of light with high and low correlated color temperature [53]. This phenomenon probably reflects difficulties in subjectively comparing the brightness of light stimuli of different colors [60]. In addition, it has been shown that brightness perception has a short-wavelength spectral sensitivity that increases with increasing light levels [61], which could explain the greater brightness perception of the short wavelength light in our study. The evaluation of short-wavelength light as more activating corroborates the alerting and performance enhancing effects of short-wavelength light.

In terms of visual comfort, the increase in HES symptoms during the shift suggests that neither the short-wavelength nor the long-wavelength light was ideal, although there was improved visual comfort with short-wavelength light. However, we cannot discern between these effects being related to the light conditions alone or the night work itself. Still, the evidently reduced symptoms with short-wavelength light compared to long-wavelength light, and the relatively large amount of explained variance for some of the symptoms (e.g., eye fatigue), suggests that the light conditions are of importance. Note that compared to HES symptoms reported during daytime office hours [51], the severity for most symptoms in the current study was considerably larger in both light conditions in the middle and later parts of the night shift. The previous study of office workers did not investigate narrow-bandwidth light but compared strongly blue-enriched light (17,000 K) with standard white light (4000 K) [51]. Improved visual comfort with blue-enriched light was found among the office workers [51] and this was suggested to be related to pupil responses driven by melanopsin and the ipRGCs [13], and it was further suggested that greater pupil constriction may have contributed to the improved visual comfort. Similar effects may explain the findings in the current study as the short-wavelength light triggered a far higher melanopsin stimulation than the long-wavelength light. Recently, it was suggested that the mechanisms for light exacerbating migraine headaches can be explained by responses of cone driven retinal pathways [62]. In the current study, both the short-wavelength and long-wavelength light stimulated the cones, but the long-wavelength light had more than twice the L-cone-opic (i.e., long wavelength cone) irradiance than the short-wavelength light, with 38.3 and 14.6 µW/cm^2^, respectively. Hence, this difference may also contribute to the increased HES symptoms experienced with the long-wavelength light.

In the present study, subjective measures and task performance assessments were repeated throughout the night shift in order to investigate the effects of short-wavelength and long-wavelength narrow-bandwidth light on participants’ functioning. A crossover design was used to control for interindividual differences in the dependent variables and interindividual variability in responses to light [56]. To ensure no crossover effects, counterbalancing and a four-week washout period between night shifts were applied. Unfortunately, some participants did not complete both night shifts (i.e., both light conditions). However, despite missing data, compared to many previous trials, the current study included a relatively large sample size in concordance with recent recommendations [43]. In addition, the statistical analysis strategy (using LMM and GLMM analyses) allowed for missing values in the dataset without excluding cases completely. Nevertheless, caution should be taken, particularly when interpreting the circadian phase shift data, as there was a relatively large amount of missing DLMO estimates. Participants were not extreme chronotypes, not allowed to nap, and were selected based on sleep timing and duration criteria. Bearing in mind the use of a crossover design, differences in homeostatic sleep pressure likely did not have significant impact on the results. It should be noted that the current sample consisted of relatively young adults. Thus, it is not clear whether the current results can be generalized to other populations and age groups. It is known that with older age there are retinal changes, i.e., yellowing of the lens, and light exposure can differentially impact nonvisual responses during extended wakefulness in young and older individuals [63]. In addition, since males have shown greater nonvisual responses to light and differ in their opinion of lighting compared to females [64], a sample with a more even sex distribution could have given rise to somewhat different results.

The present study demonstrates a novel use of ceiling mounted LED-luminaires for administering narrow-bandwidth light conditions during simulated night work. Furthermore, we employed relatively high photon densities of light, which may be realistic for real-life work situations. However, the practical relevance for night workers remains debatable. Ambient narrow-bandwidth lighting alters visibility and color rendering (i.e., color appearance of the surroundings), hence for many workplaces the narrow-bandwidth lighting employed in the present study may not be feasible. The novel way of administering the narrow-bandwidth light conditions in the current study, however, offers new opportunities for illumination that need further investigation in terms of feasibility for specific workplaces and settings. A concern with short-wavelength light, in particular, relates to the potential negative impact of light at night [34,35], e.g., melatonin suppression and circadian disturbance. The present results indicate strong phase shifting effects of the short-wavelength light. While such effects may be practical for permanent night workers, they may at the same time be regarded as unwanted effects for rotating night workers. Another consideration, not assessed in the present paper, is the impact of light interventions on sleep and recovery after night work, as sleep disturbances are one of the main issues with night work [24,25]. Thus, there is a need to consider which effects of light are most desired in specific work situations and settings, particularly for night workers.

We did not thoroughly control participants’ light exposure prior to the night shifts, as has been done in previous laboratory studies. Light exposure in the hours (18:00–22:45) preceding the night shifts was monitored by the light sensor on the actigraph device, indicating no significant difference between conditions. Prior light history is known to affect nonvisual light responses, including the alerting response [65]; hence, this may have also had an impact in the current study. However, the relatively high latitude and time of year ensured limited daylight exposure in the hours preceding the night shifts. Furthermore, we did not employ individually tailored light exposure, as suggested by a recent study [30]. Due to the inter-individual differences in circadian phase timing, the fixed work schedule, and uniformly lit work environment, the light exposure occurred at different circadian times for different participants. Hence, individually tailored light exposure would likely lead to even larger phase shifting effects than observed. We aimed to keep the study similar to a real-life night work setting and put limited restraints on the participants during their spare time. It can thus be viewed as a strength that our findings were in agreement with the previous highly controlled laboratory studies showing alerting and phase shifting effects of duly timed short-wavelength narrow-bandwidth light. A limitation with the present study was that the participants did not complete a baseline test bout just prior to the light exposure. Thus, we cannot exclude that variation in the assessed parameters (PANAS, KSS, PVT, and DSST) may have existed prior to light exposure, and that the first 30 min of light exposure affected the parameters. However, considering the repeated measures design and counterbalancing, it is unlikely that this greatly distorted the results.

Future studies should investigate the possibility of providing individually tailored light exposure, using standard ceiling mounted LED-luminaires, e.g., by programming luminaires to provide favorable light exposure at individual workplaces. Furthermore, there is a need for more studies to assess the amount of melatonin suppression throughout the night shift under different light conditions.

## 5. Conclusions

The current study revealed beneficial effects of exposure to short-wavelength narrow-bandwidth light (λ_max_ = 455 nm), compared to photon matched (~2.8 × 10^14^ photons/cm^2^/s) long-wavelength narrow-bandwidth light (λ_max_ = 625 nm), on subjective alertness and task performance during a simulated night shift. Moreover, the participants’ melatonin onset was more phase delayed in short-wavelength light compared to long-wavelength light. It was demonstrated that short-wavelength narrow-bandwidth light can improve alertness and performance, as well as strengthen circadian phase shifting, during simulated night work using standard ceiling mounted LED-luminaires and relatively high light levels. Participants evaluated both light conditions as moderately pleasant and moderately suitable for work, albeit visual comfort was higher in short-wavelength light compared to long-wavelength light. These results show that standard LED-luminaires can be used to administer short-wavelength narrow-bandwidth light with the potential to improve alertness and performance among night workers. However, more studies are needed to validate these findings, e.g., in different populations, and to investigate the applicability of such light conditions in real-life workplaces. There is a need to further study LED-based lighting in order to develop lighting recommendations for night workers.

## Figures and Tables

**Table 1:** Light exposure at eye level (vertical plane) for the two light conditions.

	Short-Wavelength Narrow-Bandwidth Light	Long-Wavelength Narrow-Bandwidth Light
	Mean (SD)	Mean (SD)
Peak spectral irradiance (nm)	455	625
Irradiance (µW/cm^2^)	125.0 (5.4)	82.6 (4.9)
Photopic illuminance (lx)	60.8 (4.0)	195.9 (10.6)
Melanopic EDI (lx)	584.5 (23.0)	4.1 (1.6)
Photon density (photons/cm^2^/s)	2.9 × 10^14^ (1.4 × 10^13^)	2.6 × 10^14^ (1.5 × 10^13^)
Human photoreceptor responses (irradiance—µW/cm^2^)		
S-cone-opic	97.0 (3.9)	0.2 (0.1)
M-cone-opic	23.7 (1.0)	9.5 (0.6)
L-cone-opic	14.6 (0.8)	38.3 (2.0)
Rhodopic	63.5 (2.5)	1.3 (0.2)
Melanopic	77.5 (3.1)	0.5 (0.2)

Note: Light was measured in the beginning, middle, and end of each night shift at two desk spaces. Lighting parameters computed according to the CIE S 026 Toolbox—version 1.049 [45]. EDI = equivalent daylight illuminance.

**Table 2:** Effects of light condition and time on mood, subjective sleepiness, task performance and visual comfort.

	Long-Wavelength Light	Short-Wavelength Light	Light		Time		Light by Time		
	EMM (SE)	EMM (SE)	*F* (*df*)	*p*	*F* (*df*)	*p*	*F* (*df*)	*p*	*R* ^2^
PANAS (10–50 (extremely))									
Positive mood	19.63 (0.87)	20.22 (0.86)	1.13 (1, 278)	0.289	56.81 (4, 267)	<0.001	1.57 (4, 267)	0.182	0.28
Negative mood	11.55 (0.31)	11.74 (0.31)	0.99 (1, 278)	0.320	3.93 (4, 267)	0.004	3.24 (4, 267)	0.013	0.05
Sleepiness (KSS, 1–9 (sleepy))	6.97 (0.21)	6.28 (0.21)	25.32 (1, 278)	<0.001	59.23 (4, 266)	<0.001	3.61 (4, 266)	0.007	0.30
Psychomotor vigilance task									
Mean 1/RT	2.82 (0.08)	2.97 (0.08)	18.36 (1, 272)	<0.001	32.21 (4, 266)	<0.001	3.29 (4, 266)	0.012	0.14
Mean RT500	342.79 (5.46)	332.40 (5.44)	25.24 (1, 270)	<0.001	40.97 (4, 266)	<0.001	4.04 (4, 266)	0.003	0.14
Number of lapses (RTs ≥ 500 ms) ^a^	6.95 (1.49)	4.82 (1.09)	6.60 (1, 290)	0.011	37.41 (4, 290)	<0.001	2.68 (4, 290)	0.032	-
Digit symbol substitution test (*n* correct)	73.50 (1.32)	76.35 (1.31)	18.17 (1, 273)	<0.001	8.48 (4, 265)	<0.001	1.58 (4, 265)	0.180	0.08
Headache and eye strain scale (1–4 (severe))									
Irritability	1.71 (0.12)	1.70 (0.12)	0.01 (1, 155)	0.928	7.41 (2, 147)	0.001	2.78 (2, 147)	0.065	0.05
Headache	1.80 (0.11)	1.58 (0.11)	7.14 (1, 156)	0.008	18.45 (2, 147)	<0.001	0.57 (2, 147)	0.568	0.11
Eye strain	2.73 (0.12)	2.44 (0.12)	8.33 (1, 157)	0.004	39.45 (2, 146)	<0.001	2.08 (2, 146)	0.129	0.22
Eye discomfort	2.50 (0.14)	2.22 (0.14)	7.18 (1, 154)	0.008	29.75 (2, 146)	<0.001	3.82 (2, 146)	0.024	0.15
Eye fatigue	2.86 (0.11)	2.49 (0.11)	14.18 (1, 158)	<0.001	86.07 (2, 145)	<0.001	3.97 (2, 145)	0.021	0.39
Difficulty focusing	2.72 (0.13)	2.45 (0.13)	7.72 (1, 154)	0.006	56.07 (2, 145)	<0.001	2.05 (2, 145)	0.132	0.25
Difficulty concentrating	2.74 (0.11)	2.39 (0.11)	11.27 (1, 160)	0.001	74.03 (2, 147)	<0.001	1.45 (2, 147)	0.238	0.38
Blurred vision	2.06 (0.14)	1.82 (0.13)	6.43 (1, 154)	0.012	13.83 (2, 146)	<0.001	1.54 (2, 146)	0.219	0.07

Note: Variables analyzed using linear mixed model analyses. ^a^ Analyzed using generalized linear mixed models. *R*^2^ is the proportion of reduction in variance of the residuals between a random effects model and the interaction effects model. EMM = estimated marginal mean; SE = standard error; *df* = degrees of freedom; PANAS = Positive and Negative Affect Schedule; KSS = Karolinska Sleepiness Scale; RT = response time.

## Supplementary Materials

The following are available online at https://www.mdpi.com/2624-5175/2/4/37/s1.
