# Supplementary material for: Alerting and Circadian Effects of Short-Wavelength vs. Long-Wavelength Narrow-Bandwidth Light during a Simulated Night Shift
Source: Clocks Sleep. 2020 Nov 25;2(4):502–22. doi: 10.3390/clockssleep2040037 (PMC7712639; doi:10.3390/clockssleep2040037)
Supplement: Supplementary file 1 [file clockssleep-02-00037-s001.zip › clockssleep-938166 suppl for publish.pdf]

Table S1. Light exposure per condition in the hours (18:00–22:45) preceding the night shifts.

	**Short-Wavelength Narrow-Bandwidth Light (*n* = 27)**	**Long-Wavelength Narrow-Bandwidth Light (*n* = 28)**	***p*** [a]
	**Mean (SD)**	**Mean (SD)**	
Total white light exposure (lx/min)	17.63 (13.47)	19.65 (22.19)	0.320
Maximum white light exposure (lx)	341.22 (212.19)	431.42 (367.23)	0.495

Note. Light exposure levels assessed with wrist-actigraphy (Actiwatch 2 or Actiwatch Spectrum). [a] Analyzed using Wilcoxon signed ranks test.
